# Structural Features of Y_2_O_2_SO_4_ via DFT Calculations of Electronic and Vibrational Properties

**DOI:** 10.3390/ma14123246

**Published:** 2021-06-11

**Authors:** Aleksandr S. Oreshonkov, Yuriy G. Denisenko

**Affiliations:** 1 Laboratory of Molecular Spectroscopy, Kirensky Institute of Physics, Federal Research Center KSC SB RAS, 660036 Krasnoyarsk, Russia; 2 School of Engineering and Construction, Siberian Federal University, 660041 Krasnoyarsk, Russia; 3 Department of General and Special Chemistry, Industrial University of Tyumen, 625000 Tyumen, Russia; yu.g.denisenko@gmail.com

**Keywords:** yttrium oxysulfate, DFT, lattice dynamics, Infrared, Raman, vibrations, Y_2_O_2_SO_4_

## Abstract

The traditional way for determination of molecular groups structure in crystals is the X-Ray diffraction analysis and it is based on an estimation of the interatomic distances. Here, we report the analysis of structural units in Y_2_O_2_SO_4_ using density functional theory calculations of electronic properties, lattice dynamics and experimental vibrational spectroscopy. The Y_2_O_2_SO_4_ powder was successfully synthesized by decomposition of Y_2_(SO_4_)_3_ at high temperature. According to the electronic band structure calculations, yttrium oxysulfate is a dielectric material. The difference between the oxygen–sulfur and oxygen–yttrium bond nature in Y_2_O_2_OS_4_ was shown based on partial density of states calculations. Vibrational modes of sulfur ions and [Y_2_O_2_^2+^] chains were obtained theoretically and corresponding spectral lines observed in experimental Infrared and Raman spectra.

## 1. Introduction

The rising in experimental and theoretical studies of rare-earth-activated phosphors over the past decades is primarily associated with their applications in lighting, electronic displays, temperature sensing, etc. [1]. A large variety of rare-earth doped inorganic compounds have been synthesized, such as molybdates [2,3,4], tungstates [5,6,7], phosphates [8,9,10], aluminates [11,12,13] and silicates [14,15,16].

Since the chemical formula contains trivalent rare-earth (*Re*^3+^) ions, the common way for doping is a partial substitution of *Re*^3+^ with *Ln*^3+^ (*Ln*^3+^ = Ce, Pr, Nd, Sm, Eu, Tb, Dy, Ho, Er, Tm, Yb, Lu) ions. Recently, *Re*_2_O_2_SO_4_ oxysulfate was studied as a host for optical materials and it has been shown that the luminescent efficiency of *Re*_2_O_2_SO_4_:*Ln*^3+^ phosphorous depends on the size and shape of particles [17], for example, the Eu^3+^ doped nanosized Y_2_O_2_SO_4_ samples (18–89 nm, *C*2/*c*) show the quantum efficiencies ranging from η = 44–70% [18]. The 2–3 μm in diameter Y_2_O_2_SO_4_:Eu^3+^ was synthesized using a urea-based homogeneous precipitation technique based on a urea-ammonium sulfate system [19]. The Y_2_O_2_SO_4_:Tb^3+^ microflakes were prepared via an electrospinning process followed by calcination treatment [20]. The biomolecule-assisted hydrothermal route followed by calcination was used for the production of yttrium oxysulfate hollow spheres doped with Yb^3+^ and Eu^3+^ or Er^3+^ [21].

Traditionally, the search of nonlinear optical (NLO) materials focused on borate systems [22], on the other hand, several NLO sulfate crystals were synthesized in recent years [23,24,25,26,27]. As to the *Re*_2_O_2_SO_4_ oxysulfates, the crystal structure of Nd_2_O_2_SO_4_ [28] was solved in the non-centrosymmetric (*I*222) space group and thus this class of compounds can be a candidate for NLO materials. On the other hand, the crystal structure of Sm_2_O_2_SO_4_ [29] and Eu_2_O_2_SO_4_ [30] was solved in the centrosymmetric (*C*2/*c*) space group.

Determination of non-centrosymmetric or centrosymmetric space groups in *Re*_2_O_2_SO_4_:*Ln*^3+^ can be easily done with Infrared spectroscopy as has been shown in work by Yu.G. Denisenko [30]. The focus of such study must be pointed to the presence of the peak related to the symmetric stretching vibration of SO_4_ tetrahedra in case of the *C*2/*c* space group. However, it should be noted that while the interpretation of the spectral peaks related to vibrations of sulfate groups is beyond doubt, vibrations of rare-earth ions were explained as just Y-O vibrations and detailed description of these vibrations is completely absent in the literature. The spectral bands at 1220, 1130, 1060, 1000, 880, 660 and 610 cm^−1^ were observed in Infrared spectra of Y_2_O_2_SO_4_:Eu^3+^ and attributed to vibrations of SO_4_^2−^ ions, while the peak at 530 cm^−1^ was described as Y-O bond vibration [19]. In nanometer-sized Y_2_O_2_SO_4_:Eu^3+^, the Y-O bond peak was found at 560 cm^−1^ [31]. In Infrared spectra of Y_2_O_2_SO_4_ doped with Tb^3+^ ions, characteristic spectral bands related to sulfate vibrations and the Y-O stretching (at 539 cm^−1^) were observed [20]. Spectral peak related to the stretching vibrations of O-Y was found at 545 cm^−1^ in Y_2_O_2_SO_4_ [32]. The spectral bands in Y_2_O_2_SO_4_ nanoparticles at 1064, 1122, 133 and 664 have been attributed to SO_4_^2-^ ions while bands at 621 and 534 to Y-O vibrations [33]. There is no information at all about Raman spectra.

In this paper, we report the synthesis of Y_2_O_2_SO_4_, results of DFT (Density Functional Theory) calculations of electronic and vibrational properties and we demonstrated that spectral lines in Infrared and Raman spectra of Y_2_O_2_SO_4_ were associated with [SO_4_]^2−^ and [Y_2_O_2_^2+^] structural units.

## 2. Materials and Methods

### 2.1. Synthesis and Experimental Details

**Figure 1:**
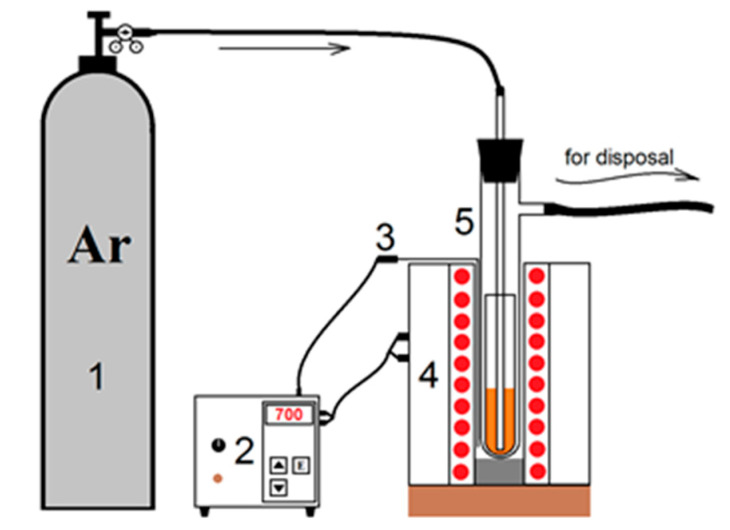
Installation for the synthesis of yttrium oxysulfate. 1—argon tank; 2—thermoregulator; 3—thermocouple; 4—vertical oven; 5—quartz reactor.

Yttrium oxysulfate was obtained by decomposition of yttrium sulfate Y_2_(SO_4_)_3_ (99.99%, Novosibirsk Rare Metals Plant, Novosibirsk, Russia) in an argon atmosphere at a temperature of 700 °C. A schematic of an installation for carrying out high temperature decomposition processes is shown in Figure 1. Argon of high purity 99.9999% was used to create an inert atmosphere. Temperature control and regulation was carried out using a microprocessor controller (“Thermokeramika”, Moscow, Russia). Temperature in the reaction zone was measured with a chromel-alumel thermocouple. A weighed portion of dry Y_2_(SO_4_)_3_ was placed in a quartz reactor and purged with argon for 30 min at a rate of 6 L/h. After that, the reactor was placed in a heated vertical furnace and held for 10 h. After the completion of the reduction process, the reactor was removed from the furnace and cooled to room temperature. The decomposition recovery process is described by the equation:

Y_2_(SO_4_)_3_ → Y_2_O_2_SO_4_ + 2SO_2_ + O_2_

Fourier-transformed Infrared spectroscopy (IR) was carried out with the use of a Fourier Transform Infrared Spectrometer FSM 1201, (Infraspec Ltd., Borovliany, Minsk district, Belarus). The sample for the investigation was prepared in a tablet form with addition of annealed KBr. IR spectrum was recorded with spectral resolution 4 cm^−1^. Raman spectrum was recorded using an i-Raman Plus spectrometer at a laser excitation wavelength of 785 nm and the spectral resolution was about 4 cm^−1^. The Infrared as well as the Raman spectrum was obtained at room temperature.

### 2.2. Calculation Details

All the density functional theory calculations [34,35] were performed with the CASTEP code (version 19.1.1) [36]. The 4s^2^4p^6^4d^1^5s^2^, 3s^2^3p^4^ and 2s^2^2p^4^ valence electron configurations were used for Y, S and O, respectively. The local density approximation (LDA) based on the Perdew and Zunger parametrization [37] of the numerical results of Ceperley and Alder [38], and nonlocal exchange-correlation HSE06 functional [39] were used for calculation of electronic properties. The on-the-fly-generated norm-conserving pseudopotentials were used and the cutoff energy for the plane basis was chosen as 1150 eV. The convergence criteria for geometry optimization were set to 5.0 × 10^−4^ eV/Å for maximal force and 0.01 GPa for maximal stress. The density functional perturbation theory (DFPT) (linear response method) [40] was used to perform the calculation of vibrational properties. Different k-point density [41] was checked for Monkhorst–Pack sampling [42] and it was found that the 6 × 6 × 3 k-point set is enough.

## 3. Results and Discussion

**Figure 2:**
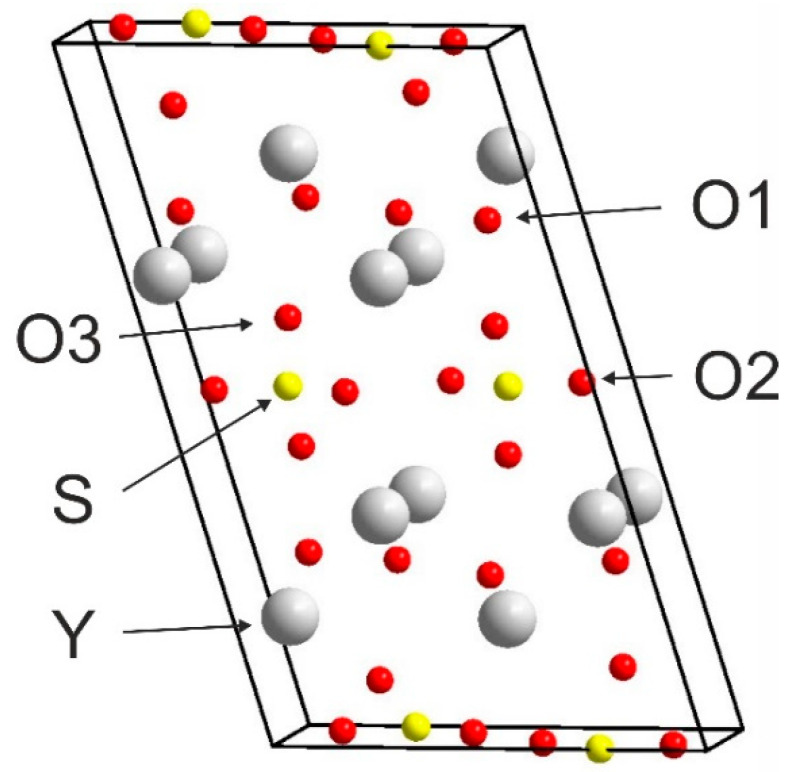
Conventional cell of Y_2_O_2_SO_4_.

Figure 2 presents the crystal structure of Y_2_O_2_SO_4_. Investigated sulfate presents a monoclinic structure with the *C*2/*c* space group (#15). As can be seen from Figure 2, the crystal structure consists of SO_4_ layers and layers formed with Y and O ions. Calculated values of lattice parameters and atomic coordinates are presented in Table 1 and compared with experimental data from ICDD PDF 53-0168.

**Figure 3:**
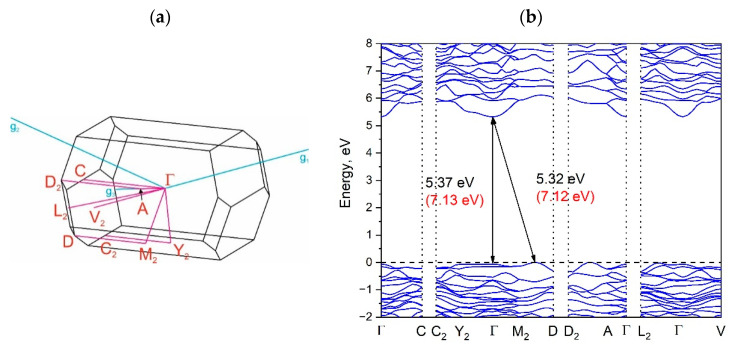
Brillouin zone (**a**) and electronic band structure (**b**) of Y_2_O_2_SO_4_. Band gap values obtained with the hybrid functional are shown in parentheses in (**b**).

**Figure 4:**
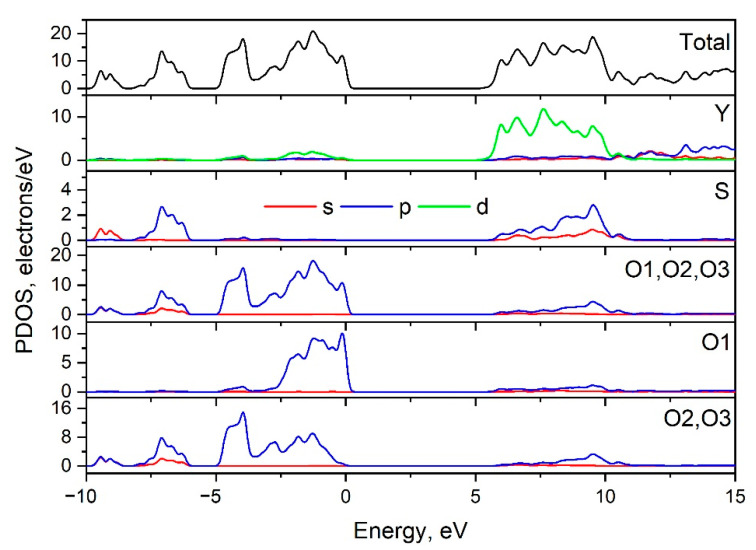
Total and partial density of electronic states of Y_2_O_2_SO_4_.

**Figure 5:**
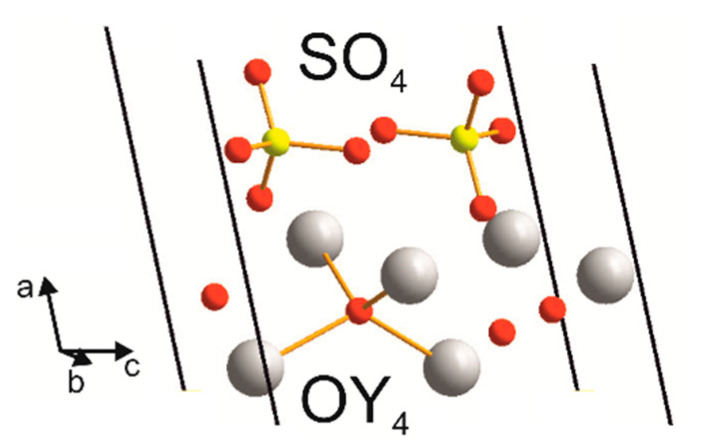
*AB*_4_ (*A* = S, O; *B* = O, Y) structural units in Y_2_O_2_SO_4_.

The Brillouin zone (BZ) of Y_2_O_2_SO_4_ and electronic band structure obtained using the local density approximation are shown in Figure 3. The path along high symmetry points of BZ was selected as: Γ–C|C_2_–Y_2_–Γ–M_2_–D|D_2_–A–Γ|L_2_–Γ–V_2_. Coordinates of these points are: Γ(0, 0, 0), C(−0.276, 0.276, 0), C_2_(−0.724, −0.276, 0), Y_2_(−0.5, −0.5, 0), M_2_(−0.5, −0.5, 0.5), D(−0.737, −0.263, 0.5), D_2_(−0.263, 0.263, 0), A(0, 0, 0.5), L_2_(−0.5, 0, 0.5), V_2_(−0.5, 0, 0) (Figure 3a). The conduction band minimum (CBM) is located at the Γ point, while the valence band maximum (VBM) is located between M_2_ and D k-point, thus making the Y_2_O_2_SO_4_ an indirect band gap material, the E_g_^i^ = 5.32 eV (Figure 3b). However, the difference between indirect and direct electronic transition is small, the value of the calculated direct band gap is 5.37 eV. Taking into account that the experimental band gap value of Y_2_O_2_SO_4_ has not yet been published and DFT calculations in LDA approximation generate a band structure which underestimates the gap [43], the electronic band structure was calculated using the HSE06 hybrid functional. The value of indirect and direct electronic transition obtained with HSE06 are 7.126 and 7.131 eV correspondingly. In the meantime, the electronic density of states (DOS) and partial DOS are shown in Figure 4. It is clearly seen from Figure 4 that the top of valence band is formed by p-electron of oxygen, while the bottom of the conduction band comprises Y’s d-electrons. It is interesting to note that partial densities of states are different for oxygen ions in SO_4_ tetrahedra and O1 ions located between Y layers (see Figure 2) and the VBM is formed with O1 atoms. Thus, the wide band gap dielectric behaviors (E_g_(HSE06) = 7.12 eV) of Y_2_O_2_SO_4_ are connected with the structural layer formed with Y and O1 atoms. The electronic density of states of O2 and O3 atoms (in SO_4_ tetrahedra) from −8 to −6 eV has contribution from 2s and 2p orbitals while the same region is empty in DOS of O1. We suppose that, in this case, the DOS of O1 in the range of −2.5–0 eV corresponds to the hybrid sp orbital, see Figure 4, and the OY_4_ molecule can be distinguished as a separate structural unit, see Figure 5.

**Figure 6:**
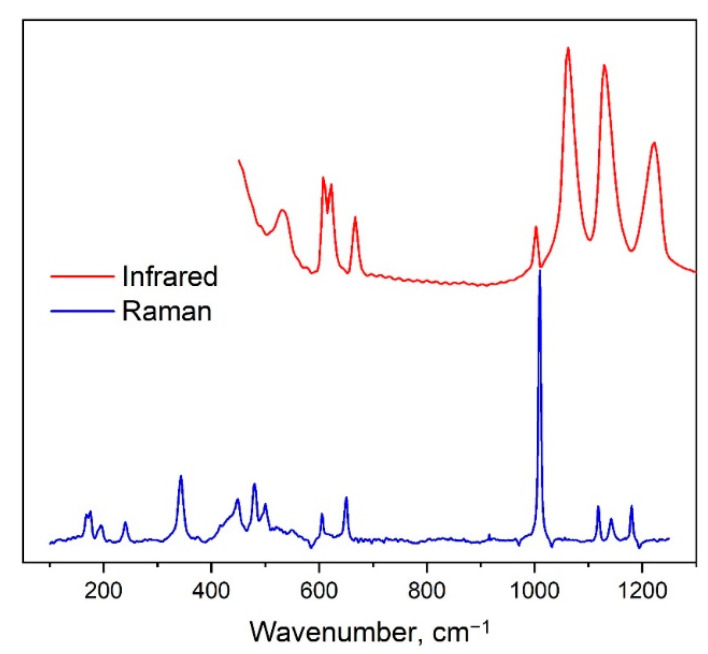
Experimental Infrared and Raman spectra of Y_2_O_2_SO_4_.

The Y_2_O_2_SO_4_ belongs to the monoclinic space group with the factor group symmetry C^6^_2h_. Vibrational representation for the yttrium oxysulfate at the center of the Brillouin zone can be written as follow: Γ_vibr_ = 13*A_g_* + 13*A_u_* + 14*B_g_* + 14*B_u_*. The *A_u_* + 2*B_u_* are acoustical translational modes while the remaining *A_u_* and *B_u_* modes are Infrared-active, the *A_g_* and *B_g_* are Raman-active vibrations. In the structure of Y_2_O_2_SO_4_, the SO_4_ tetrahedra occupy the positions with *C*_2_ symmetry and relation between free [SO_4_]^2−^ ion with *T_d_* symmetry, its site symmetry and the factor group symmetry of the monoclinic cell are presented in Table 2. According to Table 2, nine internal vibrations of SO_4_ should be observed in Raman as in Infrared spectra. The Infrared and Raman spectra of Y_2_O_2_SO_4_ are presented in Figure 6. The total set of observed spectral lines, DFT calculated wavenumbers and mode assignments are presented in Table 3.

The weak spectral band at 969 cm^−1^ in the Infrared spectrum and strongest band at 1009 cm^−1^ in the Raman spectrum are associated with ν_1_ symmetric stretching vibrations of SO_4_ groups. Spectral bands above 1000 cm^−1^ are antisymmetric stretching vibrations (ν_3_). Bands at 650 and 604 cm^−1^ in Raman spectrum are ν_4_ antisymmetric bending of SO_4_ tetrahedra. The ν_4_ modes appeared in the Infrared spectrum at 666, 621 and 608 cm^−1^. Spectral lines in Raman spectrum at 448 and 432 cm^−1^ are defined as ν_2_ symmetric bending of sulfur ions. The rotational vibration of SO_4_ appeared in Raman spectrum as the peak at 195 cm^−1^. The Raman peak at 168 cm^−1^ is explained as translations of SO_4_ in the SO_4_ structural layer.

**Figure 7:**
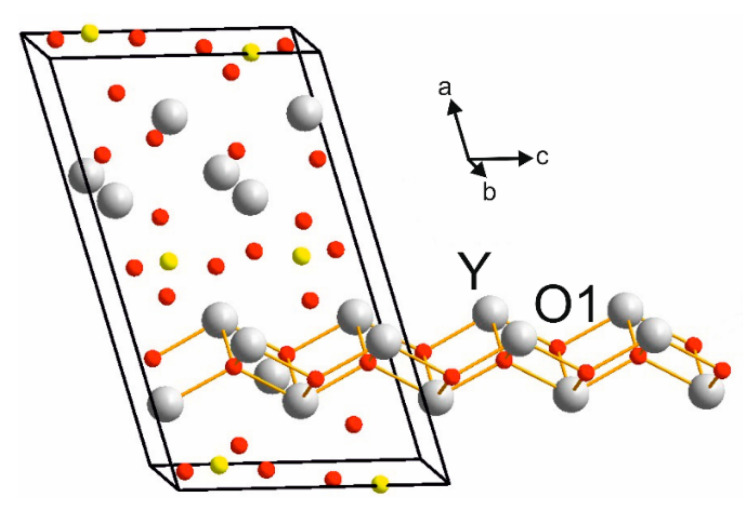
Representation of [Y_2_O_2_^2+^] layers in crystal structure of Y_2_O_2_SO_4_.

**Figure 8:**
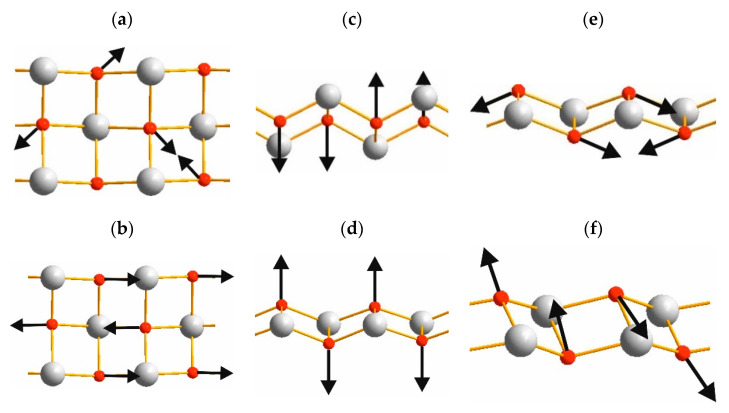
Representative atomic vibrations of [Y_2_O_2_^2+^] chains in Y_2_O_2_SO_4_: (**a**) *B_g_* 533.1 cm^−1^, (**b**) *A_g_* 505.3 cm^−1^, (**c**) *B_g_* 383.2 cm^−1^, (**d**) *A_g_* 352.5 cm^−1^, (**e**) *A_u_* 546.7 cm^−1^, (**f**) *B_u_* 499.1 cm^–1^.

Connection of the OY_4_ tetrahedra (Figure 5) into the chains creates the [Y_2_O_2_^2+^] layers as shown in Figure 7 and vibrations of these layers have been found in Raman spectra. Spectral band at 500 cm^−1^ is related to O-O stretching, as shown in Figure 8a. The line at 480 cm^−1^ is an antiphase translation of O atoms along the layer, Figure 8b. The spectral line at 374 cm^−1^ is an antiphase vibration of O in [Y_2_O_2_^2+^] structural units as shown in Figure 8c. The strong band at 344 cm^−1^ is an antiphase vibration of O atoms, Figure 8d. The *A_u_* mode (Infrared active) with a calculated wavenumber value equal to 546.7 cm^−1^ is a combination of ν_2_ vibrations of SO_4_ and O-O stretching, as shown in Figure 8e. The *B_u_* mode at 499.1 cm^−1^ is a translation of O, as shown in Figure 8f. Thus, we can conclude that the wide spectral band at 532 cm^−1^ in Infrared spectra is devoted to oxygen vibration in [Y_2_O_2_^2+^] chains, but not to Y-O vibrations as was stated earlier [19,20,31,32,33]. The assignment of remain vibrational modes is presented in Table 3.

## 4. Conclusions

In summary, we have demonstrated that the lines in vibrational spectra of Y_2_O_2_SO_4_ should be interpreted in terms of vibrations of SO_4_ tetrahedra and layers composed of [Y_2_O_2_^2+^] structural units. Calculated partial density of states shows different electron distribution for s and p orbitals in case of oxygen atoms in [SO_4_]^2−^ and in case of [Y_2_O_2_^2+^]. The formation of hybrid sp orbital in yttrium–oxygen chains is supposed. The electronic structure and band gap value of yttrium oxysulfate was presented for the first time.

## Figures and Tables

**Table 1:** DFT calculated structural parameters for Y_2_O_2_SO_4_ in comparison to ICDD PDF 53-0168.

Lattice Dimensions, Å	a	b	c
Calc. (this work)	13.1242	4.0956	7.8734
ICDD PDF 53-0168	13.3076	4.1465	8.0204
**Lattice Angles, Degrees**	**α, γ**	**β**	
Calc. (this work)	90	107.292	
ICDD PDF 53-0168	90	107.64	
**Fractional Coordinates**	**x**	**y**	**z**
Y	0.17153	0.48979	0.08471
O1	0.24427	0.97988	0.12243
O2	0.9997	0.26714	0.09789
O3	0.09659	0.84802	0.29921
S	0	0.05146	0.25

**Table 2:** Correlation between internal vibrations of SO_4_ tetrahedra in Y_2_O_2_SO_4_.

Wavenumber, cm^−1^ [44]	*T_d_*	*C*_2_	*C*_2*h*_
983	*A*_1_ (ν_1_)	*A*	*A_g_* + *A_u_*
450	*E* (ν_2_)	2*A*	2*A_g_* + 2*A_u_*
1105	*F*_2_ (ν_3_)	*A*+2*B*	*A_g_* + *A_u_* + 2*B_g_* + 2*B_u_*
611	*F*_2_ (ν_4_)	*A*+2*B*	*A_g_* + *A_u_* + 2*B_g_* + 2*B_u_*

**Table 3:** Calculated and experimental phonon wavenumbers (cm^−1^) of Y_2_O_2_SO_4_ with proposed assignments. Notations: Irreps.—irreducible representations, str.—stretching, tr.—translation, rot.—rotation, def.—deformation.

Infrared				Raman			
*Irreps*.	Calc	Exp	Assignment	Irrep*s*.	Calc	Exp	Assignment
*B_u_*	1155.6	1219	ν_3_ SO_4_	*B_g_*	1154.3	1180	ν_3_ SO_4_
*A_u_*	1095.9	1133		*B_g_*	1123.3	1142	
*B_u_*	1030.4	1063		*A_g_*	1096.3	1118	
*A_u_*	969.0	1002	ν_1_ SO_4_	*A_g_*	980.0	1009	ν_1_ SO_4_
*B_u_*	636.0	666	ν_4_ SO_4_	*B_g_*	632.0	651	ν_4_ SO_4_
*B_u_*	592.1	621		*B_g_*	629.3	648	
*A_u_*	581.9	608		*A_g_*	584.3	604	
*A_u_*	546.7		ν_2_ SO4 + O1-O1 str.	*B_g_*	533.1	500	O1-O1 str.
*B_u_*	499.1	532	O1 tr.	*A_g_*	505.3	480	O1 tr.
*A_u_*	481.7		ν_2_ SO_4_ + O1-O1 str.	*B_g_*	492.9	448	O1-O1 str.
*A_u_*	461.9		ν_2_ SO_4_	*A_g_*	492.6	448	ν_2_ SO_4_
*A_u_*	429.2		O1 tr.	*A_g_*	476.2	432	ν_2_ SO_4_
*B_u_*	416.3		O1 tr.	*A_g_*	453.6	416	ν_2_ SO_4_ + O1-O1 str.
*B_u_*	378.8		O1 tr.	*B_g_*	383.2	374	O1-O1 tr.
*A_u_*	334.3		O1 tr.	*A_g_*	352.5	344	O1-O1 tr.
*A_u_*	260.3		Y tr.	*B_g_*	275.5		SO_4_ def.
*B_u_*	231.2		SO_4_ rot.	*A_g_*	248.3	240	Y tr.
*B_u_*	216.2		SO_4_ rot.	*B_g_*	240.2		Y tr.
*A_u_*	191.3		SO_4_ rot.	*B_g_*	207.7		SO_4_ tr.
*B_u_*	187.4		SO_4_ tr.	*B_g_*	203.1		SO_4_ tr.
*B_u_*	157.7		SO4 tr.	*A_g_*	202.1	195	SO_4_ rot.
*A_u_*	151.6		SO4 def.	*A_g_*	188.8		SO4 tr.
*B_u_*	131.6		SO_4_ tr.	*B_g_*	184.7		SO4 rot. + tr.
*A_u_*	96.7		Y tr.	*A_g_*	172.5	172	Y tr.
				*B_g_*	161.2	168	SO_4_ tr.
				*A_g_*	151.9		Y tr.
				*B_g_*	120.0		SO_4_ tr. + [Y_2_O_2_^2+^] tr.

## Data Availability

The data presented in this study are available on request from the corresponding author.
